# Forecast Modelling via Variations in Binary Image-Encoded Information Exploited by Deep Learning Neural Networks

**DOI:** 10.1371/journal.pone.0157028

**Published:** 2016-06-09

**Authors:** Da Liu, Ming Xu, Dongxiao Niu, Shoukai Wang, Sai Liang

**Affiliations:** 1 School of Economics and Management, North China Electric Power University, Beijing, China; 2 School of Natural Resources and Environment, University of Michigan, Ann Arbor, MI, United States of America; Jiangnan University, CHINA

## Abstract

Traditional forecasting models fit a function approximation from dependent invariables to independent variables. However, they usually get into trouble when date are presented in various formats, such as text, voice and image. This study proposes a novel image-encoded forecasting method that input and output binary digital two-dimensional (2D) images are transformed from decimal data. Omitting any data analysis or cleansing steps for simplicity, all raw variables were selected and converted to binary digital images as the input of a deep learning model, convolutional neural network (CNN). Using shared weights, pooling and multiple-layer back-propagation techniques, the CNN was adopted to locate the nexus among variations in local binary digital images. Due to the computing capability that was originally developed for binary digital bitmap manipulation, this model has significant potential for forecasting with vast volume of data. The model was validated by a power loads predicting dataset from the Global Energy Forecasting Competition 2012.

## Introduction

Forecasting is critical for the social and engineering sciences. Future trends can usually be predicted by a numerical model given available information[1]. The majority of conventional forecasting models (usually refers to regression models), including statistical models and machine learning models, are designed to approximate the functional relationships among variables. Quantitative variables, which are usually expressed in decimal format, are efficiently addressed in these models, whereas categorical data are seldom involved in the models. However the rapid development of information and communications technology has increased the availability of new information in various formats other than only quantitative variables, such as text, voice and image[2]. The data in these new forms contain information that is increasingly important for forecasting, nevertheless they are hard to be analysed by functional approximation that used in the conventional regression models. Thus, the complete and successful exploitation of the data in these formats is a major challenge for conventional forecasting models.

In fact, all these data are stored and processed in a uniform binary digital format in computers. If we can directly model variables in binary digital format to find their relationship instead of decimal format, discrepancies among data formats will cause fewer problems.

One consideration in this approach is the efficient processing of binary digital data. Progress in image processing techniques has provided an abundance of high-performance algorithms that can run on fast graphics processing units (GPUs) and highly optimized implementations of two-dimensional (2D) convolutions[3–5]. Thus, we need to translate the data to binary digit format (if they are in decimal or other formats) and represent the data using 2D images for computer processing. This approach enables even a micro-change of a variable, whether categorical or quantitative, to be detected based on the disparity among binary images. To construct forecasting models with complex relationships among the variables, we can construct a network using convolutional computing and scaling calculation techniques that are extensively applied in the field of image recognition to mine the local connection of binary digits from dependent variables to independent variables.

Deep learning (DL) neural networks exhibit striking capabilities in the field of image recognition[6–11]. Using multi-layer hierarchical network structures, DLs automatically extract meaningful features of data from layer to layer, significantly improving the classification performance of machine learning applications[12]. These techniques remove the limitations of conventional artificial intelligence applications that are substantially dependent on domain expertise to select the proper input and parameters. A convolutional neural network (CNN), which is a specific type of DL model, abstracts local and basic features from lower layers to higher layers to complete a classification task via pooling and convolutional computing[13]. A CNN distinguishes itself from conventional machine learning models by exhibiting dramatic improvements in recognition applications[14]. However, CNNs have been primarily applied to classification tasks.

We propose to employ CNNs for forecasting as an alternate to regression techniques. The advantages of using a CNN-based forecasting model with binary digital image input are as follows:

1. When represented in binary digital form, all inputs and outputs, including categorical variables and/or any other variable formats such as images, can be easily processed by the model;
2. Instead of mining the relationships between the variables using an approximation function, which is employed in conventional forecasting, it mines the relationships between the original variables in binary digit form from local viewpoints; and
3. Binary digital information can be efficiently processed using high-performance GPUs and 2D convolutions that have been successfully adopted in image recognition.

In this paper, we apply CNNs to an experiment that involves power load forecasting, as this is a common problem in the forecasting field[15–19]. The fluctuation in power loads correlates to a variety of factors, including historical loads, time-dependent variables such as weekdays and holidays, and weather conditions such as temperature[20]. Historical loads and temperature are quantitative data that can be easily addressed; however, calendar variables that provide time-dependent information about historical loads and temperatures are categorical information that poses challenges for traditional forecasting techniques.

We employ a dataset that is provided by Global Energy Forecasting Competition 2012 (GEFCom 2012)[15] to demonstrate our approach. This dataset provides hourly loads for 20 different zones and hourly temperatures for 11 weather stations from Jan. 1, 2004, to June 20, 2008. However, history loads of eight specific weeks are missing and are set to be backcasted, and the hourly loads for the first seven days of July 2008 are expected to be forecasted even though the temperatures for those days are unknown.

## Methods

### CNN for Classification and Regression

A CNN is a biologically inspired network with alternating convolutional and subsampling layers that are employed to draw local information from lower levels to higher levels. A CNN has a fully connected layer that is employed to generate outputs. Using local connections, shared weights, subsampling, and sparse connectivity, a CNN can be trained to achieve remarkable performance levels in many applications[14]. The objective of both regression and classification is to determine a mapping relationship between dependent variables and independent variables[21]. This study proposes a method to bridge the gap between classification and regression by transforming maximum probability labelling to a weighted sum. The basic input units for an image recognition task are pixels, which are easily represented by binary vectors. For a conventional forecasting task, however, the input variables are in decimal format and cannot be represented in pixels for local connection computing. If we reorganize the original variables via multiple independent units (e.g., binary digits), in which each unit represents local information of the original real variables—similar to pixels in image recognition tasks—we can obtain a 2D digital image, which is the same input/output form that is employed for CNN image recognition models. After obtaining the binary digital outputs of a CNN, we can perform the forecasting by reconverting these outputs to decimal form.

### Data

**Fig 1:**
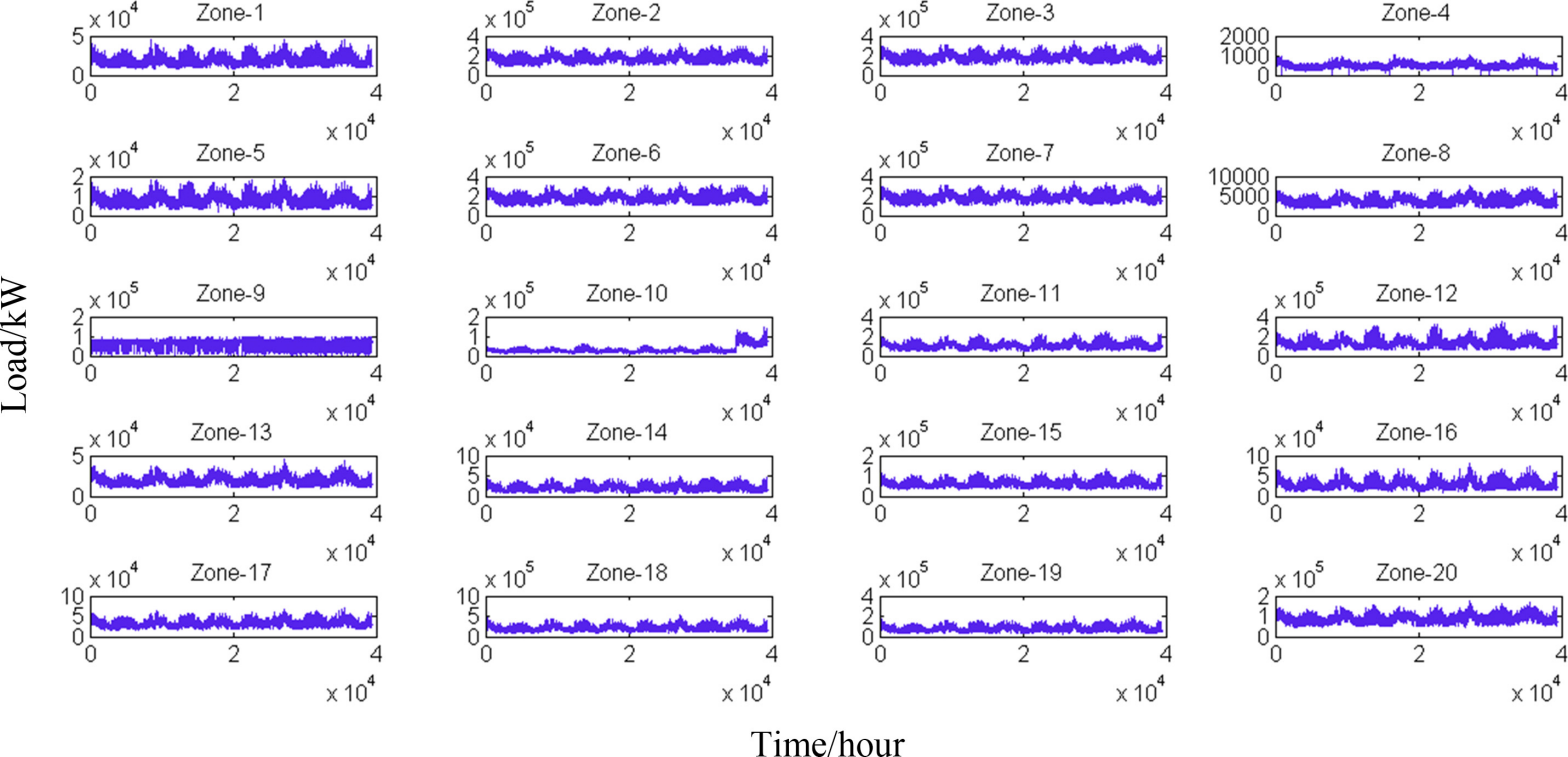
Historical loads in the 20 zones. The loads in the majority of the zones are approximately measured at the 100,000 kW level; however, some zones have loads near the 10,000 kW level, such as Zone 1, Zone 13 and Zone 14. Zone 4 has the lowest load of less than 1,000 kW. Large differences in the load fluctuations of these 20 zones are observed.

The data applied to demonstrate the methodology are obtained from GEFCom 2012[15]. This dataset includes the historical loads of 20 zones (Fig 1) and historical temperatures from 11 associated weather stations. Loads in the different zones significantly vary; therefore, we separately model the load for each zone. As the load consistently shows strong hourly fluctuations[22], 24 models are constructed; one model is constructed for every hour. A total of 480 models are constructed for the forecasting task. We employ the forecasting model “at Hour *h* for Zone *i*” as an example to demonstrate the modelling process.

The load data from Jan. 1, 2004, to June 30, 2008 (with the exception of the eight weeks of missing data that was previously mentioned) are applied to train the models. The missing loads for the eight weeks are to be backcasted, whereas the loads from July 1, 2008, to July 7, 2008, are to be forecasted. The temperatures for the eight backcasted weeks are available, whereas the temperatures for the hours to be forecasted in July are unknown.

### Input for Modelling

We employ the forecasting model “at Hour *h* for Zone *i*” as an example to demonstrate the modelling process. All calendar variables are selected as the first components of the input (i.e., 11 binary digit bits, which represent the four original variables). The temperature at each of the 11 stations at the forecasting hour and the temperature of the same hour of the same weekday from the previous two weeks are employed as the second components of the input (i.e., 231 binary digit bits represent the 33 original variables). Historical loads at the same hour of the same weekdays from the previous two weeks are employed as the last component of the input (i.e., 28 binary digit bits represent the two original variables). A total of 270 binary digit bits, which represent 39 original variables, are employed for the model input.

### Temperature Forecasting

As the current temperature is employed as part of the input, we have to replenish unknown temperatures when forecasting the load for the days (07/01/2008–07/07/2008). We employ historical temperatures as substitutes for the unknown temperatures. By comparing the temperature in June 2008 with the temperature of previous years, we discover that the temperature are similar to the temperature of 2005 for Station 1, Station 2, Station 4, Station 7, Station 9, and Station 11. Therefore, we employ the same temperature from 2005 to substitute for the unknown temperature of these stations. For the remaining stations, the temperature are assumed to be the same temperatures from 2007.

### Data Pre-Processing

All the input/output data must be presented in binary image format for the CNN forecasting modelling. The input variables include calendar information (year, month, day and holiday), the historical hourly loads of the Zone *i*, and the historical temperatures of 11 stations that encompass the 20 zones. The output is the load of that need to be forecasted at Hour *h* for Zone *i*. The main task in this part is to convert data from the decimal format to binary digit format then finally to binary image. Eight steps consist of the converting. Here we take the converting of the input as an example, and the output can be processed in the same way.

Step 1: Sort every variable with a certain sequence, even if it is a category type variable.
Step 2: If numerical variable, scale it to [0, 1], otherwise skip this step.
Step 3: For every variable, determine the number of bits *n*_*i*_ of variable *v*_*i*_ according to its relative importance in the input.
Step 4: Map all values of variable *v*_*i*_ to the range of [1, 2^ *n*_*i*_ -1], the first value in the sequence is set to 1, and the last value in the sequence is set to 2^ *n*_*i*_ -1.
Step 5: Convert the mapping values of variable (in decimal format) to binary format.
Step 6: Reorganize the input (consists of the decimal variables) as a binary vector that obtained in Step 5.
Step 7: Reshape the binary input vector to a certain data matrix.
Step 8: Represent the data matrix with black-white image (black for 1 and white for 0, similar to Quick Response Code in information process field).

The outline of converting original decimal input/output to binary digit format is shown in Table 1. The year variable is converted to binary format using three bits; e.g., 2004 becomes 001, and 2005 becomes 010. The month is converted to binary format using four bits; e.g., Jan. becomes 0001, and Dec. becomes 1100. We use weekday information rather than day of the month. Three binary bits are used to depict the weekday variable, where Monday is 001. One binary bit is used to indicate holidays, i.e., 1 for holidays and 0 for non-holidays. The load values are converted to 14 binary bits. They are scaled to the integers fall in the range of [1, 2^14^−1], and then transformed to binary format using 14 bits. In this manner, the minimum load corresponds to digits of 00000000000001, and the maximum load corresponds to 1111111111111. The temperature values are converted to seven binary bits, where the lowest temperature corresponds to 0000001 and the highest temperature corresponds to 1111111.

**Table 1:** Variables and their binary representations.

Variable	Original range	Binary digit range	Binary bits
Year	2004–2008	[001, 101]	3
Month	Jan.- Dec.	[0001, 1100]	4
Weekday	Mon.—Sun.	[001, 111]	3
Holiday	{Yes; No}	{1; 0}	1
Load	[Minimum, Maximum]	[00000000000001, 11111111111111]	14
Temperature	[Minimum, Maximum]	[0000001, 1111111]	7

The original variable values are represented in binary format for modelling. And in CNN forecasting modelling, the input/output are represented in binary image format.

### Parameters in the CNN

Table 2 lists the major parameters of the CNN modelling.

**Table 2:** Parameters in CNN modelling.

Parameter	Value
Layers of the CNN	5
Learning rate	1
Batch size in training	730
Epochs	250
Feature output maps in Layer 2	2
Kernel size in Layer 2	3
Pooling size in Layer 3	1
Feature output maps in Layer 4	2
Kernel size in Layer 4	4
Pooling size in Layer 5	1

## Results

### Input Pre-Processing

**Fig 2:**
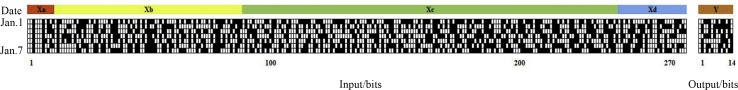
Inputs and outputs for forecasting the load at 1:00 from Jan. 1 to Jan. 7, 2004, for Zone 1 (in binary format). The top of the figure denotes the category of the input/output of the model: Xa denotes the calendar information (year, month, holiday, and weekday), Xb denotes the temperature, Xc denotes the historical temperature, Xd denotes the historical load, and Y denotes the output.

**Fig 3:**
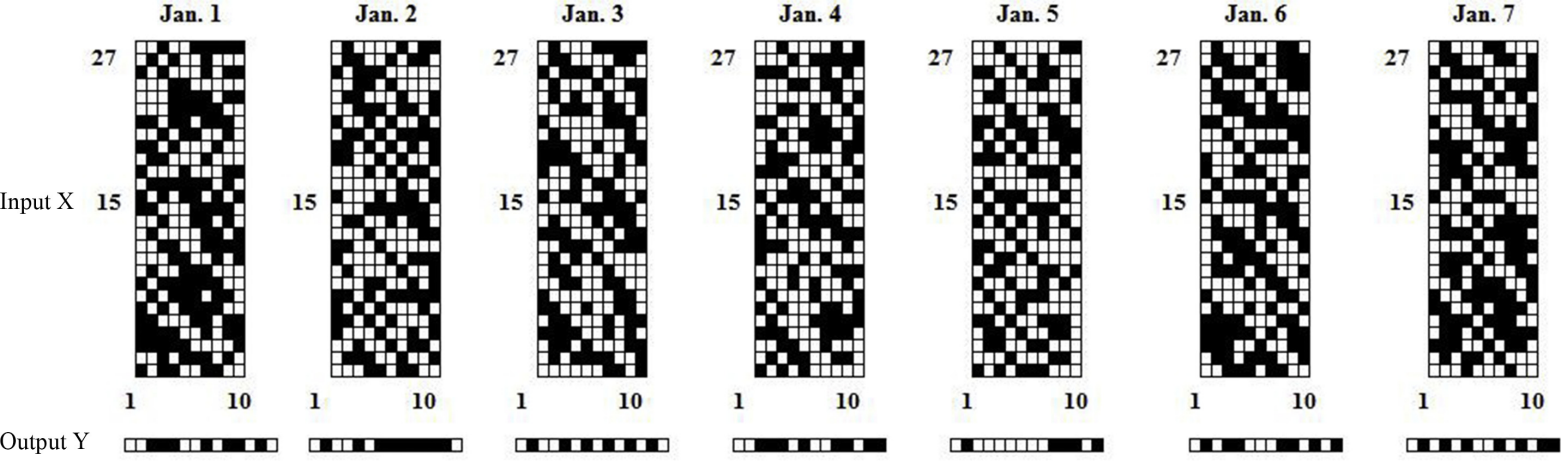
Inputs (in binary 27 × 10 image format) and outputs (in binary 1 × 14 image format) for forecasting the load at 1:00 from Jan. 1 to Jan. 7, 2004, for Zone 1. The black-and-white images in the top row are the inputs for the CNN modelling. These digital images represent binary matrices (27 × 10) that are converted from 270-bit input vectors in Fig 2. The images on the bottom row that resemble black-and-white bar codes comprise the output from the CNN and represent load output predictions in the form of 14-bit binary vectors.

Fig 2 shows the input and output in binary format for modelling the power load at 1:00 from Jan. 1, 2007, to Jan. 7, 2004, for Zone 1. Fig 3 shows the same input and output in the form of binary digit images.

### Training Error

A total of 1,460 samples are available to train each model, and 730 samples are employed for training in each batch; thus, the model is trained twice for every epoch. We train the model for 500 iterations for a total of 250 training epochs. The training root-mean-square error (RMSE) is applied to evaluate the performance of the model training, as measured in kW.

**Fig 4:**
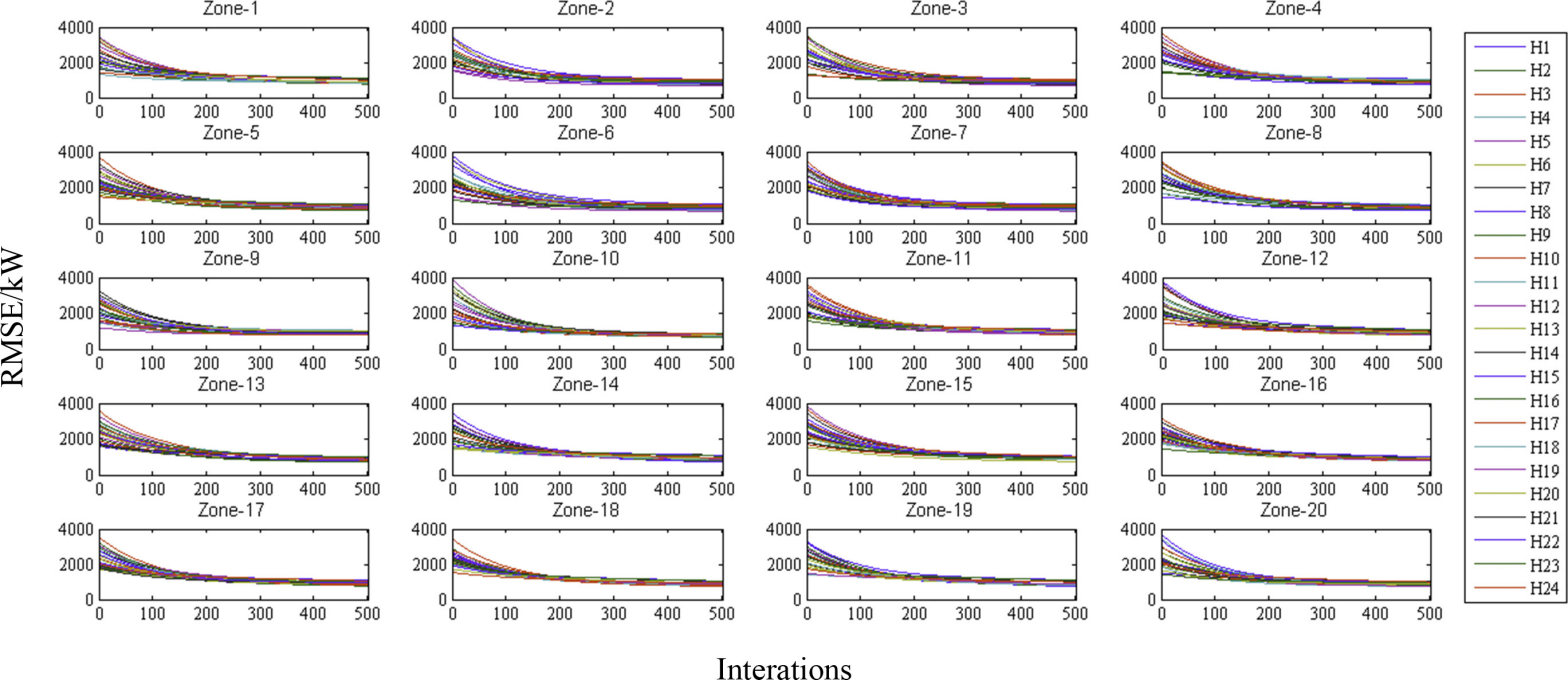
Training errors (RMSE) of 24-hour models for all 20 zones at different iterations. The sub-figures (labelled Zone-1 to Zone-20) represent the RMSE for each zone for the 24 models trained over 24 hours. The training errors for the different hours (H1 to H24) are shown in different colours. The same colours and symbol codes are applied to each zone.

Training error plots for all 24-hour models for the 20 zones are shown in Fig 4. We can see that all errors rapidly decrease over the first 200 iterations and become relatively stable after approximately 400 iterations. Therefore, 400 iterations are sufficient for training the models in this task.

### Backcasting and Forecasting

**Fig 5:**
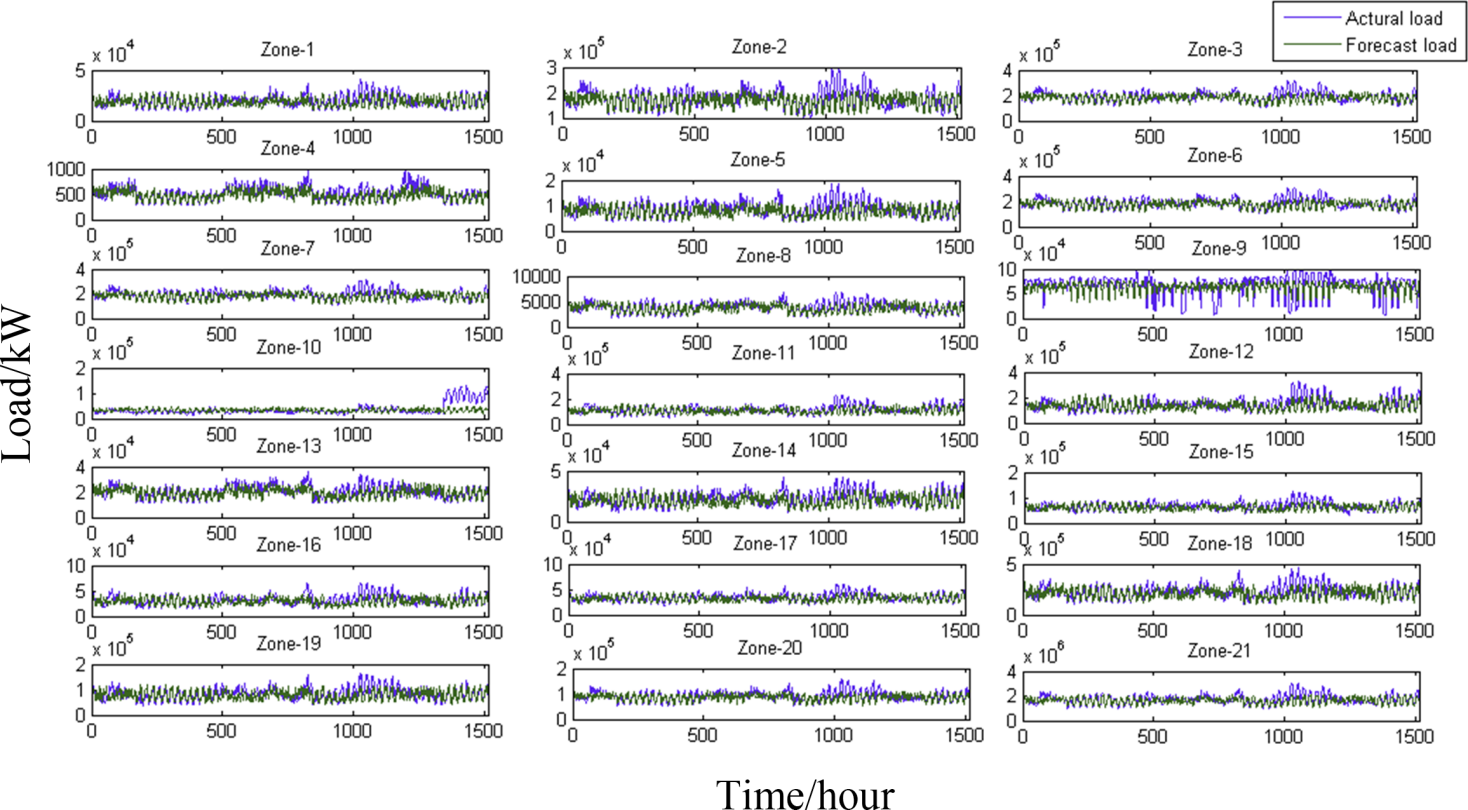
Actual and backcasted/forecasted loads for the 20 zones and the total load. The sub-figures (labelled Zone-1 to Zone-21) represent Zone 1 to Zone 20 and the total load measured in kW. The actual power load and the modelled load (including the backcasted load for the eight missing weeks and the forecasted load for one week) are shown, where blue indicates the actual load and green indicates the modelled load. The same colours and symbol codes are applied to each zone and to the total load (denoted by Zone 21).

The modelled loads (backcasted for the missing eight weeks and forecasted for the forthcoming week in July) of the 20 zones and the total loads are displayed in Fig 5. The loads are predicted with high accuracy. However, the backcasted loads for all zones after hour 1,000 are substantially lower than the actual loads. The actual loads and temperatures for May 25, 2006, to May 31, 2006, are substantially higher than the actual loads of the previous week, which likely explains why the models fail to accurately backcast the loads during this period. The performance of Zone 9 is not satisfactory due to irregular load fluctuations.

### Forecasting Evaluation

Weighted root-mean-square errors (WRMSEs) are employed to evaluate the performance of the submitted models in GEFCom 2012

WRSME=∑iwi∑i(wiAi−Pi)2∑iwi (1)

where *w*_*i*_ is the weight of the zone, *A*_*i*_ is the actual load, and *P*_*i*_ is the forecasting load.

Our model outperforms all GEFCom 2012 winners that are listed on the website of Kaggle[15] with a WRMSE of 21,709 kW for the backcast weeks and a WRMSE of 16,053 kW for the forecast week; these values are much lower than the values for the best WRMSE in the competition, which were 61,890 and 67,215 kW[15]. Therefore, the results demonstrate the significant potential of CNNs for forecasting tasks.

## Discussion and Conclusions

A forecasting model based on a CNN that employs digital images composed of binary representations of variables is proposed and compared with the winners of GEFCom 2012. This model attempts to mine mapping relationships from the internal bit changes between two variables using their local connections, as the variations in decimal values can be expressed as subtle changes in binary digits.

A successful CNN forecasting model can be constructed without any domain expertise with the proposed methodology. The implementation of the proposed modelling process is easier than the implementation of conventional methods as minimal data analysis[23] and no data cleansing process[18, 24] are required. The model provides excellent forecasting with all temperatures that are selected as input variables from the 11 stations without analysing the correlation between the station temperatures and the zone loads. The model extracts valuable information from the variables, disregards valueless information and reduces input redundancy.

The CNN forecasting model is more useful than conventional models when data are unstructured, as it successfully handles all variable types, including categorical calendar variables. By transforming the original decimal values of the variables into binary digital image form, the CNN model can automatically mine the relationship between the input and the output using local connections between binary digits. As all information, such as text, voice, and images that are stored and processed in computers, is in binary format, we expect that CNNs can be extensively applied in the future forecasting fields. The proposed methodology is successfully applied to forecast the full load electrical power output of a base load operated combined cycle power plant[25] and, showing significant performance improvement from the comparing model, which is been submitted to the same journal.

As this is the first attempt to construct a forecasting model with binary image input using a CNN, additional research may needed. A large volume of data is needed to train the CNN model to determine its connection weights. However, the suitable volume for training a specific CNN is unknown. Similar to other types of neural networks, the performance of a CNN is impacted by its parameters. Challenges such as determining the optimum set of parameters and selecting the appropriate number of bits to assign to each input/output variable represent interesting avenues for future research.
